# Workload in the Austrian IT-sector regarding leadership roles

**DOI:** 10.3389/fsoc.2024.1414420

**Published:** 2025-01-16

**Authors:** Maria Gren

**Affiliations:** Sigmund Freud University Vienna, Faculty of Psychotherapy Science, Vienna, Austria

**Keywords:** workload impact, personnel responsibility, Austrian IT sector, occupational health, Copenhagen Psychosocial Questionnaire (COPSOQ)

## Abstract

This paper investigates the impact of workload on leadership roles within the Austrian IT sector, by also paying attention to differences between genders. The research adopted a prospective design, selecting IT professionals, stratified by those with and without personnel responsibility and examined further through the lens of gender. A total of 200 participants completed the survey, where the modified German version of the Copenhagen Psychosocial Questionnaire (COPSOQ) served as the primary tool, which evaluated dimensions such as demands, influence, interpersonal relations, work interface, and conflicts. The results indicate that individuals with personnel responsibility experience significantly lower scores in the dimensions *demands* and *influence*, suggesting challenges in managing qualitative or emotional demands alongside perceived limitations in their scope of action. This trend persisted, albeit less marked, within the dimension *work interface*, indicating concerns regarding occupational stability (job security) among leading individuals. Gender analysis revealed that male participants reported fewer conflicts compared to females, highlighting discordance regarding experiencing workplace challenges. Discussion revolves around the difficulties faced by individuals with personnel responsibility in managing multifaceted demands of their role and the specific challenges encountered by female leaders. The findings emphasize the necessity of strategies to support leaders on acknowledging gender-specific challenges to enhance occupational health in the IT sector. This study contributes to the understanding of workload dynamics within leadership roles in the IT industry, recommending targeted measures to address the particular stress factors of leaders and highlight the need for gender-specific considerations in organizational support systems.

## 1 Introduction

The current technology-driven age has given the information technology (IT) sector a central role in driving innovation and economic growth. Along with this progress comes the workload of individuals with personnel responsibility, which has a significant impact on organizational health and individual wellbeing. IT leaders are at the forefront of an industry characterized by relentless pace, continuous change and the demand for constant vigilance and adaptability (Sazzad et al., 2021; Tummers and Knies, 2015). Modern leadership, particularly in IT, demands a unique blend of technical expertise, emotional intelligence, and strategic foresight. Leaders are not only expected to stay ahead of rapid technological advancements but also to foster inclusive and innovative workplace cultures. Moreover, the ability to make agile decisions under pressure while maintaining a long-term vision is increasingly seen as a hallmark of effective leadership. This specificity is further compounded by the need to inspire diverse teams, manage generational differences in workstyles, and address the unique challenges posed by hybrid and remote work environments. Recent studies reveal that the IT business report high stress levels due to workload demands, tight deadlines, and rapid technology changes. This is compounded by the cognitive, emotional, and strategic demands placed on IT professionals, especially leaders, who must balance immediate technical challenges with the foresight needed to plan the future (Shipman et al., 2023). These conditions contribute to an environment where careful examination of the impact of workload is not only beneficial, but essential to the sustainability of individuals with personnel responsibility and, by extension, the industry at large.

Research into workload in the IT industry reveals a complex set of factors that go far beyond the conventional measures of hours worked or tasks completed. They include the intricacies of cognitive demands, the emotional work involved in managing teams and the constant race to keep up with the latest technologies. The consequences of neglecting these dimensions are complex. Recent studies emphasize that cognitive overload in IT roles, often driven by the simultaneous management of multiple projects and decision-making under time pressure, significantly increases the risk of burnout and stress-related disorders (Sabharwal and Gupta, 2024). Emotional labor, particularly for IT leaders, involves navigating team dynamics, resolving conflicts, and maintaining a supportive work environment, which further amplifies workload stress and are associated with negative consequences such as burnout, reduced job satisfaction and mental exhaustion (Baeriswyl et al., 2017; Schaufeli and Bakker, 2004). At an organizational level, these factors contribute to critical challenges such as reduced employee engagement, higher turnover rates, and a deterioration of corporate culture, undermining both productivity and innovation (Gomez and Bernet, 2019). For an industry as dynamic and influential as IT, these are not just inconveniences, but fundamental challenges that can hinder progress and innovation.

The constant evolution of the IT landscape also means that the nature of the workload itself is changing. The cognitive load associated with managing constant change—be it in the form of applying new programs, new emerging data protection policies, incorporating artificial intelligence, cloud and cybersecurity developments or changing market dynamics—is significant. Research has highlighted that IT professionals increasingly report technology fatigue caused by the relentless pace of innovation and the expectation to master new tools and systems within compressed timelines (Tu et al., 2020). IT leaders, in particular, must navigate the dual demands of immediate technical problem-solving and long-term strategic planning, both of which require high levels of adaptability and resilience. This environment creates a heightened risk of decision fatigue, where the quality of leadership decisions deteriorates under sustained pressure (Madrid et al., 2019). Support mechanisms, such as regular training, mentoring programs, and resilience-building initiatives, have been shown to mitigate these effects and enable IT leaders to manage these pressures effectively (Gagnon and Monties, 2023). Organizations should prioritize these strategies to address the implications of high workloads and safeguard the health and productivity of their workforce.

A deeper dive into the nuances of workload in IT leadership reveals a complex interplay of factors where gender dynamics often exert a subtle but significant influence. Cultural stereotypes can lead to different expectations for male and female leaders, which can have an impact on everything from task assignment to performance evaluation. Women in IT leadership positions may face additional layers of complexity as they must demonstrate competency in a traditionally male-dominated field and adhere to normative gender roles that may not align with the assertive qualities typically associated with a leadership position. These inequalities require careful consideration of how workloads are distributed and perceived, and how support systems can be calibrated to address and mitigate gender-specific challenges (Aniţei et al., 2015; Gallego et al., 2015).

Furthermore, studies in various sectors have consistently shown (Davis, 2018; Gomez and Bernet, 2019) that diversity in leadership—not only in terms of gender, but also race, ethnicity and other demographic factors—leads to more innovative solutions and better organizational outcomes. Understanding and managing the dynamics of workload is therefore not just a matter of individual wellbeing, but also an investment in the collective intelligence and success of the IT sector.

Recognizing the importance of workload on IT leadership also aligns with global initiatives promoting work-life balance and mental health in the workplace. The World Health Organization (2022) has acknowledged workplace stress as a global occupational health risk, and addressing this within the IT sector is particularly pertinent given the sector’s influence on the global economy. Forward-thinking organizations are increasingly adopting policies that recognize the multifaceted nature of workload, integrating psychological support, flexible working arrangements, and proactive health and wellness programs into their corporate culture.

In light of these considerations, this paper aims to investigate the workload dynamics of IT professionals with and without personnel responsibilities, with a specific focus on gender differences. The research is therefore guided by the following questions: How do workload dimensions differ between individuals with and without personnel responsibility? To what extent do gender dynamics influence these experiences?

By addressing these questions, this paper aims to illuminate the challenges IT leaders face and contribute to the development of more resilient, adaptable, and human leadership models that are able to steer the IT sector through the challenges of the 21st century and beyond.

## 2 Method

The primary research question guiding this study is: How do workload dimensions, as assessed by the modified Copenhagen Questionnaire (COPSOQ), differ between individuals with and without personnel responsibility, and how these experiences vary by gender? This aligns with the study’s broader aim of examining the interplay between workload and leadership roles, particularly within the context of the IT sector. Based on existing literature, it is hypothesized that individuals with personnel responsibility will report greater challenges in managing demands, lower perceptions of influence and increased conflict. Furthermore, it is hypothesized that female leaders will experience higher workload-related stress compared to their male counterparts, reflecting broader gender disparities documented in IT leadership roles.

The study was designed as a prospective investigation within a sample population from the IT industry.

Where participants were recruited using a stratified sampling strategy to ensure representation across personnel responsibility levels and gender. Recruitment was conducted through professional IT networks and direct invitations to IT companies operating within Austria. Inclusion criteria were: (1) employment in the IT sector (2) residency in Austria (3) proficiency in German (to complete the questionnaire), and (4) a willingness to participate in the study. Exclusion criteria included non-IT-professionals, freelancers without organization affiliations and participants under the age of 18. These criteria ensured a homogenous sample reflecting the targeted population.

Gender was additionally incorporated as a stratified variable rather than a main factor. In the preliminary phase, sample size estimation was conducted using the G*Power^®^ 3.1.9.6 software (Faul et al., 2007). For the comparison of two groups of equal size (allocation ratio 1:1) through the application of Student’s t-test, the calculated optimal sample size was established at 150 participants, presuming a moderate yet statistically significant effect size of *d* ~ 0.50 (η^2^ = 0.06).

During the survey, a total of 260 individuals initiated the questionnaire. However, attrition occurred with 56 participants (21.5%) discontinuing their participation, and an additional four respondents were disqualified due to completing the questionnaire anomalously swiftly (in under two minutes), necessitating their exclusion from further analysis. This resulted in a final sample size of *n* = 200. Completion time of the survey instrument was a median of 7:15 (IQR 5:16; 10:04) minutes for the 200 partakers. Participants were allocated into two different groups based on their responsibility status (no responsibility *n* = 126, with responsibility *n* = 74, allocation ratio 1.7: 1) and the participant’s gender was considered as stratification. For the given sample size, the power analysis demonstrated a small effect according to Cohen’s classification of *d* = 0.41 (η^2^ = 0.04), which reaches significance (Cohen, 1988).

### 2.1 Data management

The data was converted from Lime-Survey into a SPSS matrix. Descriptive and inferential statistical analyses were performed using IBM SPSS^®^ 27.0.1. The significance level, according to the type 1 error, was set at *α* = 5% so that a result with *p* ≤ 0.05 is designated as significant in the inferential section in the statistics. In order to express the practical relevance of significant results, effect sizes according to Cohen’s classification were calculated.

Descriptive statistics were performed using key values, such as means (*M*), standard deviations (*SD*), and/or percentages to present the characteristics of the sample. Inferential analyses were performed using two-way MANOVA to examine the effects of personnel responsibility and gender on the five COPSOQ scales. Post-hoc tests were conducted -where necessary- to identify specific group differences. The statistical methods were chosen due to their suitability for analyzing multiple dependent variables simultaneously while accounting for interactions between independent variables (responsibility x gender). The selection of MANOVA was justified by its ability to handle interdependent scales, such as demands and influence, without inflating type I error rates. Additionally, chi-squared tests were used for categorical, ensuring comprehensive exploration of group differences. Effect sizes were calculated to contextualize findings, using Cohen’s classification to distinguish between small, moderate and large effects. With the utilization of the Fisher r-to-z transformation, the webpage^1^ computed a z-value that can be utilized to evaluate the statistical significance of the disparity between two correlation coefficients, r_a_ and r_b_, discovered in two separate and unrelated samples.

### 2.2 Instruments

The modified German version of the COPSOQ was used as the primary instrument for the survey. This tool was chosen for its strong psychometric qualities, including high reliability and validity in evaluating psychosocial work factors across various industries. Its widespread use in professional settings, particularly in knowledge-intensive and high-pressure sectors, highlights its versatility. Additionally, the scales have consistently demonstrated strong internal consistency. This makes the COPSOQ particularly suitable for the IT sector, as it effectively captures the multidimensional nature of workload, lending credibility to its application here.

According to the purpose of the survey, items were removed (B1:6, B3:8, B5:6, B6:3, B6:7) as well as added after FFAW^2^ (instead of B2:3, B2:5 was placed after FFAW and instead of B2:4 the FFAW question B2:6 was inserted). The questionnaire was given online in German via Lime Survey. A total of 85 five-point (1–5) scaled questions and one 11-point (0–10) scaled item were presented. The scaling was uniform, except for one item (B12) rating format five-point Likert scale and certain items (B6:8, B6:9, B:10; B8:7 and B8:12) had to be recoded within each scale so that the direction was adjusted.

The following five areas were surveyed using the COPSOQ questionnaire:

1. Demands (German: Anforderungen).
2. Influence (German: Einfluss und Entwicklungsmöglichkeit).
3. Interpersonal relation (German: Soziale Beziehung und Führung).
4. Work interface.
5. Conflicts plus single item about the general health (0–10) (German: Beschwerden, Outcomes).

Firstly, the items in the corresponding scales were evaluated considering internal consistency. The coefficient used was Cronbach’s *α*, which treats each individual item as a separate test section (Rost, 2004). In addition, the corrected part-whole correlation was taken into account, as shown in Table 1.

**Table 1:** Characteristics of internal consistency in five scales (*n* = 200).

Scale (direction)	Items (k)	Cronbach (*α*) [95%–CI]	Item part-whole corr. (min-max)	Item part-whole corr. (*Md*)	*M* ± *SD*
Demands (+)	13	0.90 [0.87–0.92]	0.35–0.76	0.60	3.10 ± 0.76
Influence (−)	17	0.85 [0.81–0.88]	0.21–0.62	0.46	2.46 ± 0.53
Interpers. Relations (−)	23^a^	0.90 [0.89–0.92]	0.09–0.67	0.57	2.57 ± 0.59
Work interface (+)	4	0.80 [0.75–0.84]	0.53–0.70	0.61	3.79 ± 0.96
Conflicts (−)	28^b^	0.93 [0.91–0.94]	0.34–0.63	0.55	2.53 ± 0.59

a 5 items recoded.

b 11 items recoded.

The results indicate acceptable internal consistencies, so that one of the criteria for validity of the survey can be assumed.

The B:12 item on general health showed a correlation of *r* = −0.57 [−0.65; −0.46] with scale 5 (conflicts), sharing the same construct at a moderate rate (*R^2^*) of 31.9% explained variance.

Furthermore, the intercorrelations between the scales were determined to estimate their extent of independence. The calculations were performed separately for the factor *personnel responsibility*. In general, the intercorrelations between the subscales are relatively small to moderate as shown in Table 2.

**Table 2:** Intercorrelation matrix regarding Pearson’s r within the five scales considering responsibility status of participants (*n* = 126 no personnel responsibility, *n* = 74 participants with responsibility).

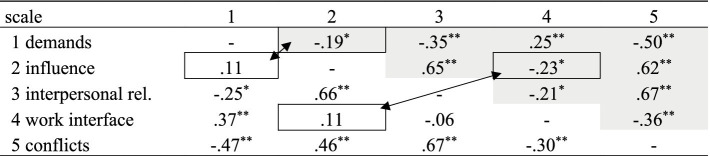

Comparisons made between two correlation coefficients using Fisher’s r-to-z transformation were additionally used to elicit and contrast the difference in correlation strengths in the two groups. Accordingly, there was a significant difference in the magnitude of the correlations for *demands* and *influence* 21–21, z = −2.04, *p* 0.041* (two-tailed) and *work interface* and *influence* 42–42 z = −2.30, *p* 0.021*. Negative relations were found for persons without personnel responsibility and positive relations for persons with personnel responsibility. This means that persons with personnel responsibility have correspondingly low values on the influence scale for the positively coded demands scale and vice versa. This pattern does not apply to persons without personnel responsibility: The response behavior shows that negative influence can occur simultaneously with high positive demands. This pattern is analogous for *work interface* and *influence*.

Regarding gender, the proportion of women in the sample was 23% of which 18.9% had personnel responsibility. The test of the distribution difference, which tested personnel responsibility in terms of gender, showed a non-significant result, χ^2^ (cF) = 1.678, *p* = 0.509, i.e., the higher proportion of men in IT typical for the industry is comparable with roughly ¾ for persons with and without personnel responsibility. Using the five-step age structure showed 8 (4.0%) of participants were between 18–24 yrs., 57 (28.5%) were between 25–34 yrs., 63 (31.5%) were between 35–44 yrs., 45 (22.5%) were between 45–54 yrs., and 27 (13.5%) ≥ 55 yrs. The comparatively most common age group was 35–44 years.

## 3 Results

This section delineates the findings pertinent to the research inquiry concerning the degree to which the five domains of workload—demands, influence, interpersonal relations, work interface, and conflicts—exhibit variation with respect to personnel responsibility and by taking gender into account.

To this end, a multivariate analysis of variance (MANOVA) was conducted on these five dependent variables, the results of which are tabulated in Table 3. The corresponding Box-M-test showed, with *p* = 0.276, homogeneity of the covariance matrices of the dependent variables across groups. Correspondingly, the nonsignificant outcomes of Levene’s tests for equality of variances across the variables affirm the assumption of homogeneity of variances.

**Table 3:** Key values (M ± SD) of workload considering personnel responsibility and gender.

Personnel responsibility	Demands (1)	Influence (2)	Interpers. rel (3)	Work interface (4)	Conflicts (5)
Male					
No (*n* = 93)	3.30 ± 0.75	2.58 ± 0.52	2.65 ± 0.60	3.85 ± 0.86	2.54 ± 0.62
Yes (*n* = 60)	2.85 ± 0.73	2.22 ± 0.48	2.40 ± 0.58	3.76 ± 1.01	2,38 ± 0.54
Total (*n* = 153)	3.12 ± 0.77	2.44 ± 0.53	2.55 ± 0.61	3.82 ± 0.92	2.48 ± 0.59
Female					
No (*n* = 32)	3.19 ± 0.73	2.57 ± 0.55	2.63 ± 0.61	3.82 ± 1.02	2.65 ± 0.56
Yes (*n* = 14)	2.71 ± 0.67	2.30 ± 0.37	2.62 ± 0.41	3.34 ± 1.18	2.71 ± 0.59
Total (*n* = 46)	3.05 ± 0.74	2.49 ± 0.52	2.63 ± 0.55	3.67 ± 1.08	2.67 ± 0.56

The individual reporting a diverse gender was kept out of the analysis (n = 199).

The results indicated no significant interactions from leadership position x gender (*p*’s ≥ 0.240), therefore the main factors could be interpreted without limitation.

### 3.1 Demands

Results showed that individuals with personnel responsibility (*M* = 2.82 ± 0.71) had significantly lower scores in the *Demands* domain compared to individuals without personnel responsibility (*M* = 3.27 ± 0.74), *F* (1, 195) = 12.30, *p* < 0.001, indicating a small effect (η^2^ = 0.059). These findings suggest that individuals in leadership roles face challenges in *Demands*, such as quantitative, cognitive, emotional and qualitative demands, compared to their counterparts without personnel responsibilities. For instance, quantitative demands often manifest as a high workload with tight deadlines, requiring leaders to manage multiple projects simultaneously while ensuring timely delivery. Cognitive demands add to this burden, as leaders are frequently required to solve complex problems, such as developing strategies to navigate sudden market shifts or resolving technical issues under pressure. Emotional demands further complicate their roles, as they must manage emotionally taxing situations, such as resolving conflicts between team members or providing support to employees facing personal or professional difficulties. Additionally, qualitative demands require leaders to maintain high standards for work quality, ensuring the precision of reports or the accuracy of strategic decisions.

### 3.2 Influence

Furthermore, it was found that persons with personnel responsibility (*M* = 2.24 ± 0.46) had significantly lower scores in the area of *Influence* compared to persons without personnel responsibility (*M* = 2.58 ± 0.53), *F* (1, 195) = 11.88, *p* < 0.001, indicating a small effect as well (η^2^ = 0.057), i.e., it can be assumed that one’s own scope of action is perceived as more limited. Individuals with responsibility can hereby perceive a restricted scope of influence over their work environment, likely due to hierarchical structures, strict company policies, budgetary or organizational constraints.

### 3.3 Interpersonal relations

No significant differences were observed in the Interpersonal Relations domain between individuals with or without personnel responsibility (*p* > 0.05).

### 3.4 Work interface

The results showed that individuals with staff responsibilities tended to have lower scores in the *work interface* domain (*M* = 3.68 ± 1.05) compared to individuals without staff responsibilities (*M* = 3.85 ± 0.86), *F* (1, 195) = 2.791, *p* = 0.096, indicating a very small effect (η^2^ = 0.014), i.e., it can be assumed that there is a trend regarding a concern about losing one’s work, being transferred or even reassigned. This reflects the precarious nature of leadership roles in the IT sector, where rapid technological changes and evolving organizational needs create uncertainty.

### 3.5 Conflicts

For the gender stratum, *Conflicts* showed a significant difference; males (2.48 ± 0.59) had lower expressions than females (2.67 ± 0.56), F (1, 195) = 4.30, *p* = 0.039, indicating a small effect (η^2^ = 0.022). Accordingly, a lower value can be found in the area of conflict among the male participants, i.e., male employees report far fewer complaints, such as exhaustion and other problems, than female employees. This disparity suggests that gendered workplace dynamics, such as stereotypes or unequal task distribution, may exacerbate workplace conflict for women.

This lower level of reported conflict among male employees suggests that gender plays a substantial role in shaping workplace experiences and perceptions of conflict. Female employees, on the other hand, appear to experience higher levels of workplace conflict, which may stem from a combination of factors rooted in gendered workplace dynamics.

One critical factor is the prevalence of stereotypes and societal expectations. Women in leadership or high-responsibility roles often face greater scrutiny and are held to higher standards compared to their male counterparts. This can lead to heightened pressure, increased emotional labor, and a greater likelihood of conflict, especially when navigating male-dominated work environments. For example, women may encounter stereotypes that question their competence or leadership abilities, which could fuel tensions in their interactions with colleagues or subordinates.

Another contributing factor is the unequal distribution of tasks and responsibilities. Research has shown that women are more likely to be assigned tasks that are seen as supportive or administrative in nature, such as organizing team events, managing interpersonal conflicts, or addressing team well-being. While these tasks are critical, they are often undervalued and add an additional layer of emotional and cognitive demands, potentially increasing the perception of workplace conflict for women (Jones and Solomon, 2019).

Moreover, the work-life balance struggle disproportionately affects women. In many cases, societal norms place a greater burden on women to manage family and caregiving responsibilities alongside their professional roles. This dual burden can lead to exhaustion and stress, which may manifest as conflict in the workplace. Female employees may also perceive workplace policies or expectations as less accommodating to their needs, which can further heighten tensions (Eagly and Carli, 2018).

These findings underline the importance of addressing structural and cultural issues within organizations. Efforts to create a more equitable workplace should focus on challenging stereotypes, ensuring fair task distribution, and fostering a culture of inclusion. Training programs aimed at unconscious bias, coupled with policies that support work-life integration, can help reduce gender-based disparities and alleviate workplace conflict for women. Expanding on these gender-related findings provides valuable insight into the unique challenges faced by women in the workplace and highlights the need for targeted interventions to foster an equitable and supportive organizational environment. This approach not only benefits female employees but also contributes to the overall health and productivity of the workplace.

**Figure 1:**
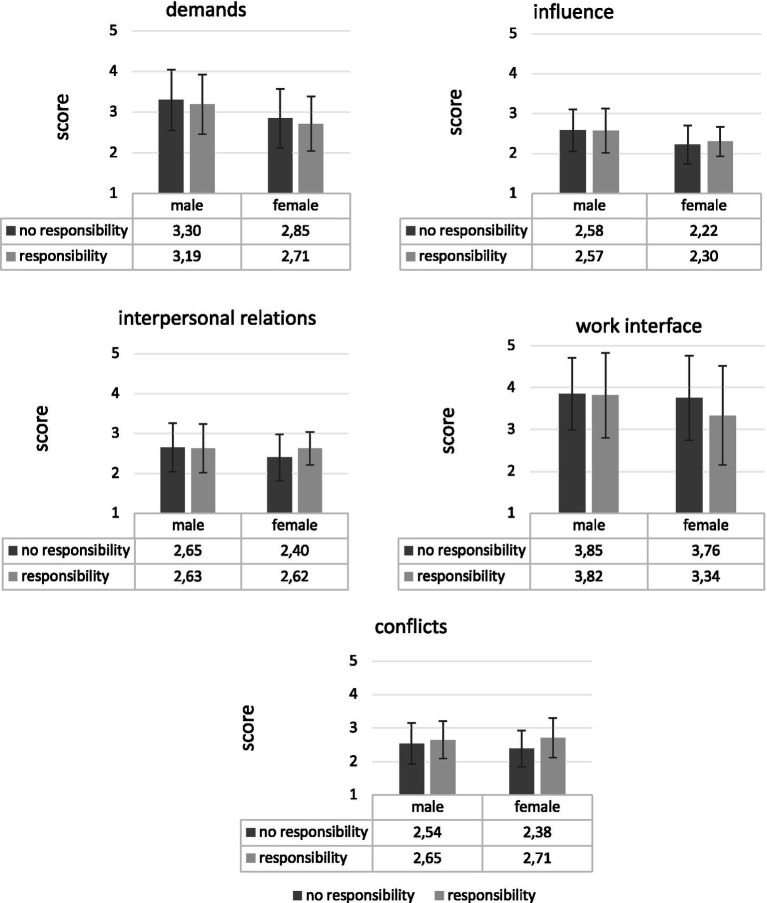
Illustrates the values of the five scales for workload as a function of personnel responsibility and gender. Key values (M ± 1 SD) of five workload scales considering participants’ gender.

All other differences regarding gender revealed no significant results (see Figure 1).

Overall, individuals with personnel responsibility faced greater challenges in managing *Demands*, *Influence* and *Work Interface* compared to their counterparts without personnel responsibility. Gender-specific findings indicated that female leaders experience disproportionately higher stress related to conflicts. These results underscore the importance of addressing workload disparities and implementing targeted support systems to enhance the well-being of IT leaders, particularly women.

## 4 Discussion

This study investigated how workload dimensions differ between IT professionals with and without personnel responsibility and explored the role of gender in shaping these experiences. The results align with the research questions and previous studies on occupational stress and workload. In particular, people with personnel responsibility show greater challenges in the dimensions:

- “*Demands*” (demands that managers have to fulfill),
- *“Influence*” (influence at work, scope for decision-making, development opportunities) and
- “*Work interfac*e” (uncertainties in relation to the workplace)

compared to their colleagues without managerial tasks.

### 4.1 Demands

Leaders reported greater challenges in managing the *Demands* dimension, particularly when trying to balance qualitative and emotional requirements alongside traditional quantitative workloads. These challenges align with previous research linking leadership roles to heightened emotional labor and stress (Baeriswyl et al., 2017; Schaufeli and Bakker, 2004). Within the “*demands*” dimension, the challenge of dealing with quantitative pressures such as looming deadlines and mandatory overtime is often exacerbated for people with leadership responsibilities. These managers are not only faced with increased quantitative demands, but also have to deal with qualitative demands such as emotional labour resulting from complex team dynamics and critical decision-making moments. Therefore, studies (Gagnon and Monties, 2023; Madrid et al., 2019) indicate the need for the promotion of emotional intelligence as well as emotional regulation in order to maintain a leader’s challenging performance and effectively manage the intricacies of their role.

Consequently, the widespread pattern of long working hours can lead to negative consequences such as chronic stress or burnout, especially when managers lack robust time management and emotion regulation strategies (Schaufeli and Bakker, 2004). This highlights the need for organizations to provide individuals with personnel responsibility with tools and training to improve their resilience and emotional intelligence to reduce the potential for work overload and the associated detriments.

Linked to the problem is often the balancing act between work commitments and personal life that leaders struggle with, and which is exacerbated by the additional responsibilities they carry. This balance is not only an individual endeavour but is also influenced by the organizational culture and support systems in place, which can either facilitate or hinder the integration of these two spheres (Greenhaus and Allen, 2011). Organizations must therefore strive to take measures and provide resources that support sustainable work-life integration, taking into account the complex interplay of individual, organizational and cultural factors.

Moreover, it is important to emphasize that a pronounced conflict between work and privacy for persons with personnel responsibility often leads to a blurring of the boundaries between their professional and personal lives—a phenomenon that has been exacerbated in the digital age, where work can invade every corner of personal space. The managerial role, with its lack of control over work factors and concern for job security, can exacerbate this conflict, leading to a constant cycle of workplace stress that spills over into personal life. This underscores the need for organizational structures that actively address work-life boundaries and provide leaders with a sense of autonomy and security in their role to promote a healthier work environment and more resilient leadership (Schaufeli and Bakker, 2004).

### 4.2 Influence

The results of this study demonstrated that individuals with personnel responsibility feel restricted in their influence. In terms of the “*influence*” dimension, a variety of factors can weaken leaders’ sense of autonomy in relation to their professional activities, particularly in relation to scheduling and staff selection. These elements manifest themselves in various organizational settings and result from the complex interplay of workplace dynamics. Rigidly defined organizational structures and deeply rooted hierarchical systems often bind persons with personnel responsibility to strict procedural rules and thus considerably restrict their freedom of action. This is particularly evident in the determination of working hours and the composition of teams. These persons who are entrusted with overseeing large areas of responsibility may lose control over individual responsibilities as the need to delegate tasks increases and comprehensive supervision decreases. Furthermore, while advances in technology have clearly improved communication channels, the prevailing expectation of constant accessibility can inadvertently limit leaders’ discretionary time management. Furthermore, in a globalized business world, individuals with responsibilities face additional obstacles, such as different time zones and different cultural practices, which further erode their control over work schedules. The complicated process of managing cross-cultural teams introduces an additional layer of complexity. Taken together, these elements lead managers to perceive their job as less impactful. Training geared towards this can produce nuanced explanations and strategies and free the individual from a perceived sense of powerlessness (RPHSR et al., 2023).

### 4.3 Work interface

Although the results regarding the differences in the work interface domain were marginal, leaders reported greater concerns about job security and organizational stability than individuals without personnel responsibility. This dimension is underpinned by existing research in the same way as the previous dimensions. For example, it has been shown that individuals with personnel responsibility struggle with job insecurity due to a variety of factors, which is extensively documented in the research (Parker, 2020) on job insecurity, stress at work and leadership challenges. The empirical research by Tu et al. (2020) distinguishes the different effects of quantitative and qualitative aspects of job insecurity on employee stress and motivation and finds that job insecurity is a key factor in occupational stress. This research describes the effects of job insecurity on employee well-being and motivation, a finding that is of particular importance for people in managerial positions in the IT environment.

One final perspective has been confirmed to have an increased relevance, namely the gender. Research in this area has highlighted inequalities in the IT sector, particularly the fact that female professionals are more likely to report health-related complaints compared to their male counterparts. It is worth noting that managing work and family commitments can lead to an imbalance in work-life balance, which particularly affects women who struggle with the challenge of balancing work and personal commitments (Byron, 2005). This conflict between work and family can in turn have a negative impact on women’s health. This insight highlights the need to take gender considerations into account when addressing occupational health and wellbeing in the IT sector.

In a broader organizational context, as Haines et al. (2016) point out, the culture of the IT industry is often permeated by gender-specific nuances. These manifest in stereotypes that can affect the wellbeing of women in predominantly male domains. Such gendered cultural dynamics exert a significant influence on the work environment and highlight the need for nuanced approaches to promote an equitable and inclusive occupational landscape.

Tying these threads together, it becomes clear that understanding the landscape of the ‘work interface’ requires recognizing the complexity of factors such as job insecurity and the multiple coping mechanisms of individuals. The interplay of these factors with the multi-layered dimensions of gender differences in the IT sector provides an overall picture that is critical to understanding the unique challenges and experiences of IT leaders, particularly as they navigate an environment that is simultaneously dynamic and characterized by traditional gender roles and expectations (Gallego et al., 2015; Haines et al., 2016).

In summary, the survey results not only confirm the trends identified in prior research regarding occupational demands but also highlight nuanced differences between individuals with and without managerial responsibilities. These distinctions have implications for workplace interventions, emphasizing the need for targeted strategies to address the unique stressors faced by managerial personnel and acknowledging gender-specific aspects in promoting occupational health within the IT industry.

Furthermore, the survey conveys a crucial narrative: the necessity to intricately weave together individual coping strategies, astute resource allocation, and the cultivation of a supportive work milieu to assuage the multifaceted challenges faced by leaders in the IT sector. By integrating these approaches, there is potential to alleviate the overall burden of workload, augmenting both individual well-being and collective team efficacy. Consequently, such integration is vital for fostering robust leadership that is equipped to navigate the complexities of the IT industry, ensuring both personal well-being and the sustainability of high-performance teams. Future research should focus on several key areas to deepen our understanding of workload dynamics in the IT sector. Investigating the long-term effects of workload disparities on IT leadership performance and retention is critical to understanding how these challenges impact both individuals and organizations over time. Additionally, exploring the effectiveness of interventions such as emotional intelligence training or participatory leadership models could provide valuable insights into strategies for reducing workload stress among leaders. Further studies should also examine the intersection of other diversity dimensions, such as race and age, to offer a more comprehensive understanding of how workload experiences differ among diverse groups in IT leadership. By addressing these research directions, future studies can build on these findings to foster healthier, more inclusive, and sustainable work environments in the IT sector.

## Data Availability

The raw data supporting the conclusions of this article will be made available by the authors, without undue reservation.

## Footnotes

^1^ http://vassarstats.net/rdiff.html

^2^Freiburger Forschungsstelle für Arbeitswissenschaften (https://www.ffaw.de/).
